# *TCF3-HLF*-Positive Acute Lymphoblastic Leukemia Resembling Burkitt Leukemia: Cell Morphologic and Immunophenotypic Findings

**DOI:** 10.1200/PO.22.00236

**Published:** 2022-08-24

**Authors:** Meng-Ju Li, Chih-Hsiang Yu, Shu-Wei Chou, Ying-Hui Su, Kuang-Wen Liao, Hsiu-Hao Chang, Yung-Li Yang

**Affiliations:** ^1^ Department of Pediatrics, National Taiwan University Hsin-Chu Hospital, Hsinchu, Taiwan; ^2^ Department of Biological Science and Technology, College of Biological Science and Technology, National Yang Ming Chiao Tung University, Hsinchu, Taiwan; ^3^ Department of Clinical Laboratory Sciences and Medical Biotechnology, College of Medicine, National Taiwan University, Taipei, Taiwan; ^4^ Department of Pediatrics, National Taiwan University Hospital and National Taiwan University College of Medicine, Taipei, Taiwan; ^5^ Childhood Cancer Foundation of the Republic of China, Taipei, Taiwan; ^6^ Institute of Molecular Medicine and Bioengineering, National Yang Ming Chiao Tung University, Hsinchu, Taiwan; ^7^ Department of Laboratory Medicine, National Taiwan University Hospital, National Taiwan University College of Medicine, Taipei, Taiwan

## Introduction

Acute lymphoblastic leukemia (ALL), the most common malignancy in childhood and adolescence, is a heterogeneous disease with various subtypes on the basis of distinct cell morphology and genetic alterations. These distinctions are noted through cytogenetic or molecular analyses and represent prognostic outcomes.^1^ By contrast, Burkitt leukemia (BL), a mature B lymphocyte neoplasm, is a highly aggressive hematologic malignancy characterized by uniformly medium-sized cells containing abundant basophilic cytoplasm, lipid vacuoles, and round nuclei with multiple nucleoli and clumped chromatin.^1^ It is associated with the proto-oncogene *MYC* at chromosomal locus 8q24.^2^

According to chromosomal analyses, approximately 80% of BL cases harbor a t(8;14) (q24;q32) translocation, which is typically used to diagnose the disease.^1^ Nonetheless, t(2;8) (p12;q24) and t(8;22) (q24;q11) may also occur.^2^ It is uncertain if true BL may be diagnosed without *MYC* translocations and hematologic cancers with BL morphology that lacked the typical BL immune phenotype or *MYC* rearrangement have been reported as BL-like ALL.^3^ Therefore, patients with leukemia with BL morphology and no *MYC* rearrangement may be diagnosed as BL-like ALL, BL without *MYC* rearrangement, or BL with cryptic *MYC* rearrangement, on the basis of next-generation sequencing.^4^ However, distinctions should be made because of differences in management and prognosis.

Because of front-line therapies derived from non-Hodgkin lymphoma Berlin-Frankfurt-Münster 95 (NHL-BFM95) protocol, pediatric BL is associated with promising outcomes. By contrast, some types of ALL with specific genetic alterations, such as t(17;19) *TCF3-HLF* fusion ALL, may harbor a dismal prognosis.^5,6^ In this study, we reported a pediatric case of *TCF3-HLF* fusion ALL wherein BL was initially diagnosed according to morphologic or immunophenotypic characteristics. The case emphasizes the importance of detecting cryptic fusion genes, especially those associated with considerable prognostic effects.

## Case Report

**FIG 1.**
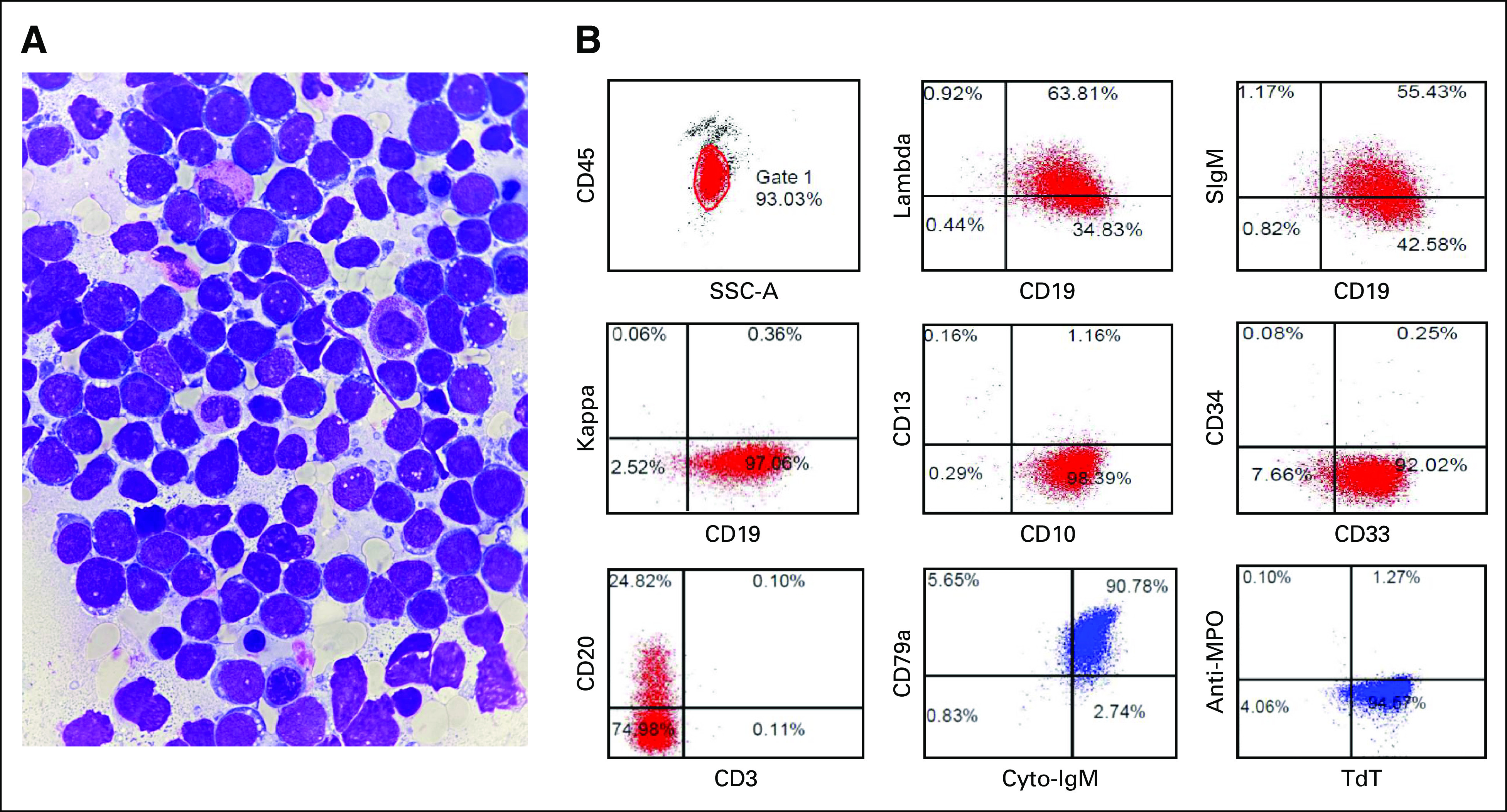
*TCF3-HLF*-positive ALL resembles Burkitt leukemia. (A) Bone marrow aspirate smear shows intermediate-sized blasts with moderately condensed chromatin, inconspicuous nucleoli, and basophilic cytoplasm with cytoplasmic vacuoles (Liu's staining; original magnification ×1,000, expanded view of the original image). (B) Immunophenotype showed positivity for CD19, CD10, CD20, CD33, CD79a, Cu, sIgM, and lambda light chain. ALL, acute lymphoblastic leukemia.

A 7-year-old boy presented with diffuse petechiae and ecchymosis. Peripheral blood exhibited WBC, 26.94 × 10^9^/L; Hb, 96 g/L; platelet, 65 ×10^9^/L; circulating blast cells, 58%; and lactate dehydrogenase, 6,000 U/L. Bone marrow aspiration identified intermediate-sized blasts with immature lacy chromatin, marked nucleoli, and basophilic cytoplasm with abundant lipid vacuoles and granules (Fig 1A). Flow cytometry of bone marrow blasts was positive for CD19, CD10, CD20, CD33, CD79a, Cu, sIgM, and lambda light chain (Fig 1B). Cytogenetic analysis revealed 46, XY, der(8), t(8:8), 19p+ [12]/49, XY, 1p+, +6, +7, 19p+, –20, +22, +mar [1]/46, XY [12]. However, t(8;14) was negative. After receiving informed consent, common fusion transcripts, including *ETV6-RUNX1*, *TCF3-PBX9*, *KMT2A* rearrangement, and *BCR-ABL1*, were screened for but not detected. The patient's bone marrow morphology and immunophenotype had overlapping features with BL. The initial CSF study showed CNS involvement.

The patient was initially treated with the Taiwan Pediatric Oncology Group (TPOG) 10 B-NHL protocol R4 arm (AA→ BB→ CC→ AA→ BB→ CC) with concurrent rituximab administration.^5,7^ Detailed medications are listed in Appendix Table A1. The bone marrow study showed complete remission after course AA. The CSF study similarly showed the absence of blasts after four courses of triple intrathecal chemotherapy (methotrexate, hydrocortisone, and cytarabine). The patient underwent sequential treatment: course BB with rituximab, course CC with rituximab, and twice triple intrathecal chemotherapy.

**FIG 2.**
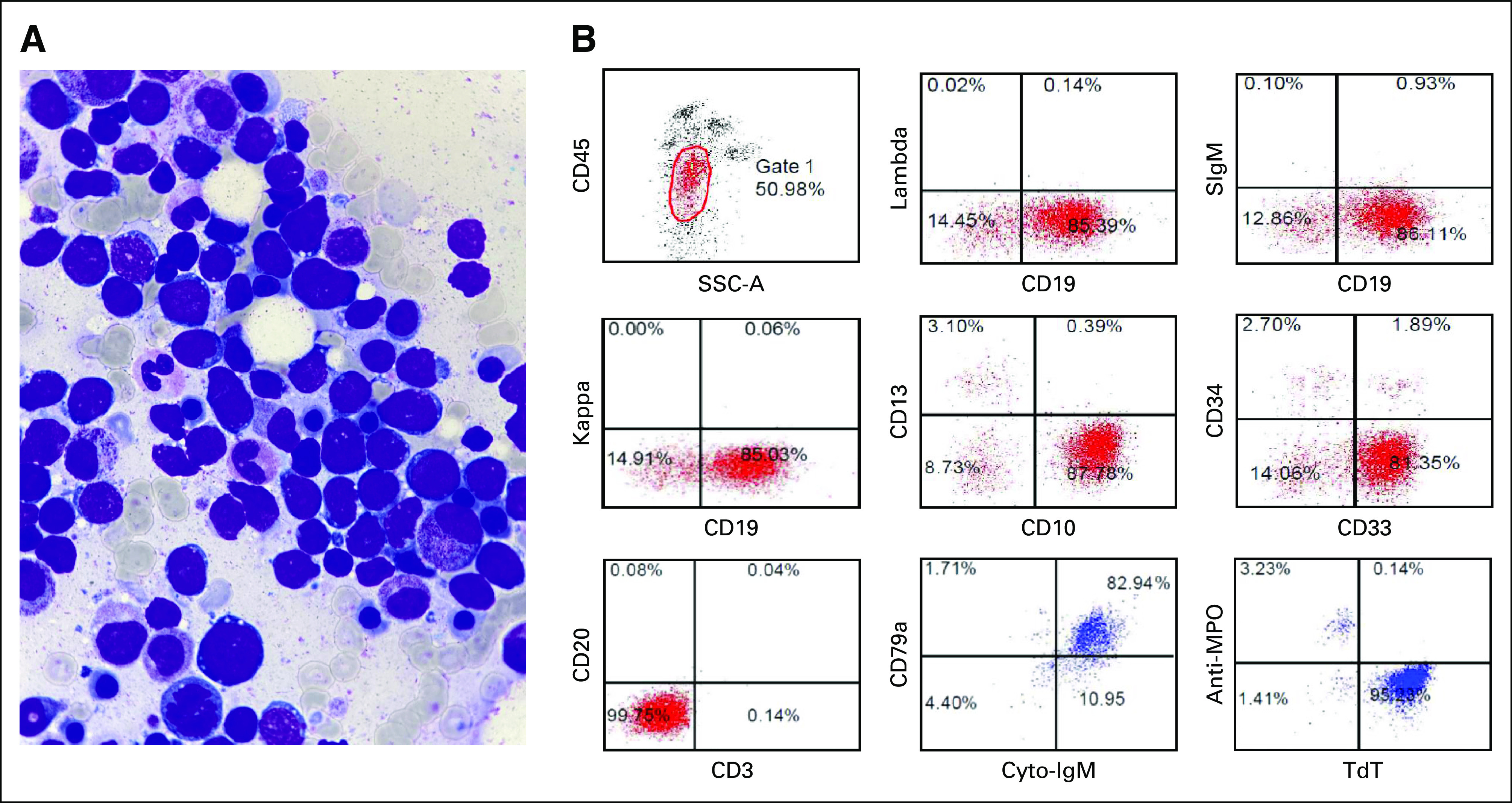
Distinct immunophenotype presentation at relapse. (A) Bone marrow aspirate smear at relapse demonstrates morphological features of BL (Liu's staining; original magnification ×1,000, expanded view of the original image). (B) Immunophenotype was positive for CD19, CD10, CD33, CD79a, and Cu, but negative for CD20, sIgM, and lambda light chain. BL, Burkitt leukemia.

**FIG 3.**
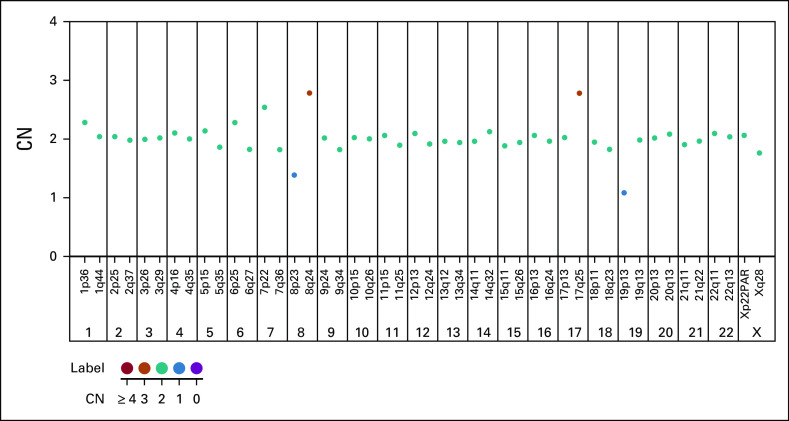
MLPA identified 19p loss. Genomic DNA was analyzed using the SALSA MLPA kit (MRC-Holland, Amsterdam, the Netherlands) according to the manufacturer's instructions, as previously described.^8^ The PCR fragments were separated by capillary electrophoresis on a Life Technologies 3500 Genetic Analyzer (Thermo Fisher Scientific, Waltham, MA). MLPA data were analyzed using Coffalyser.Net v.140721.1958 (MRC-Holland, Amsterdam, the Netherlands). A probe ratio between 0.75 and 1.3 was considered within the normal range. A probe ratio below 0.75 or above 1.3 indicated deletion or gain, respectively. A probe ratio below 0.25 or above 1.8 indicated biallelic deletion or amplification, respectively. CN, copy number; MLPA, multiplex ligation-dependent probe amplification; PCR, polymerase chain reaction.

**FIG 4.**
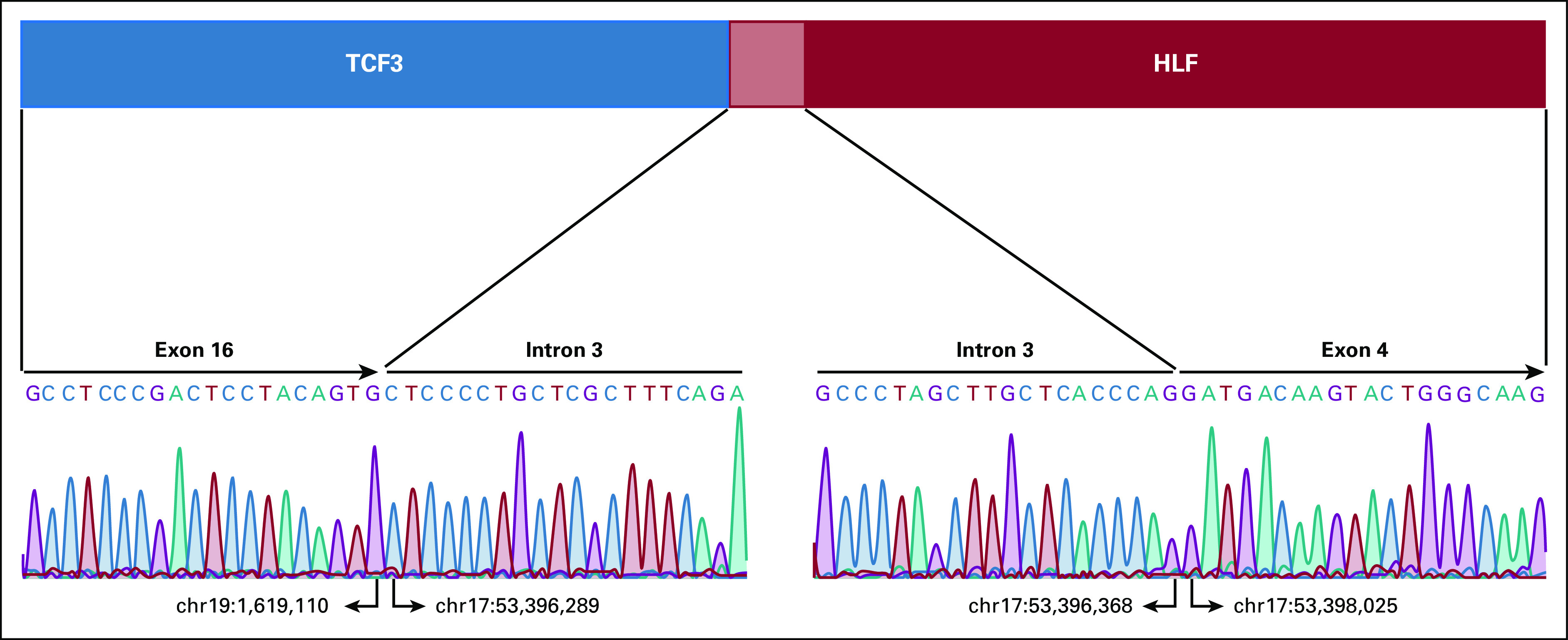
The *TCF3-HLF* fusion was verified by RT-PCR. Total RNA was isolated from bone marrow or blood samples using NucleoZol (MACHEREY-NAGEL, Dueren, Germany). Complementary DNA (cDNA) was synthesized using Maxima First Strand cDNA Synthesis Kit (Thermo Fisher Scientific, Waltham, MA). A total of 1 μg total RNA was used for cDNA synthesis, which was performed according to the manufacturer's instructions. The prepared reaction mix was incubated at 25°C for 10 minutes, followed by 60°C for 30 minutes; the reaction was then terminated by heating at 85°C for 5 minutes. PCR was performed using MyTaq HS Mix (Bioline, London, United Kingdom), and thermocycling was performed at 95°C for 60 seconds, followed by 38 cycles at 95°C for 15 seconds, 60°C for 15 seconds, and 72°C for 30 seconds. Final extension was done at 72°C for 5 minutes. Primers used for RT-PCR are sense (5′‐ GCC​TGG​CAG​GAA​CGT​CAC​AG ‐3′) and antisense (5′‐ TCA​AGT​CAG​CCA​CCT​CCT​GGC ‐3′) primers. PCR products were visualized via agarose gel electrophoresis. Suspected bands were purified using the FavorPrep GEL/PCR Purification Kit (FAVORGEN, Ping-Tung, Taiwan). Sanger sequencing was performed using an ABI 3730XL DNA analyzer (Thermo Fisher Scientific). The sequencing results were analyzed with SnapGene 4.1.3 software (GSL Biotech LLC, San Diego, CA). HLF, hepatic leukemia factor; RT-PCR, reverse-transcription polymerase chain reaction; TCF, transcription factor.

The patient completed the first round of AA, BB, and CC treatment approximately 4 months after the initial diagnosis, during which time the bone marrow study showed 40% blasts. The morphology of blasts was similar to those identified at the initial diagnosis (Fig 2A). CNS relapse was also confirmed. Flow cytometry of bone marrow blasts was positive for CD19, CD10, CD33, CD79a, and Cu, but negative for CD20, sIgM, and lambda light chain (Fig 2B). Multiplex ligation-dependent probe amplification (MLPA) detected 19p deletion, indicating TCF3 translocations (Fig 3), and *TCF3-HLF* fusion was verified using reverse-transcription polymerase chain reaction (Fig 4). Thus, the diagnosis was changed from BL to *TCF3-HLF* fusion ALL.

Seven months after the initial diagnosis, the patient had 7% residual leukemic cells detected by flow cytometry. He received haploidentical hematopoietic stem-cell transplantation sourced from the bone marrow. The conditioning regimen was total body irradiation and cyclophosphamide. Cell dosage was 9.55 × 10^6^ CD34^+^ cells/kg. The post-transplant course was uncomplicated with controllable grade II acute graft-versus-host disease. Nevertheless, a bone marrow study at 56 days post-transplantation showed 35% leukemic cells by flow cytometry, and the patient died after 2 months because of refractory disease and profound septic shock.

## Discussion

This study reports a rare case of ALL in which the blast cells expressed typical morphologic features of BL (ie, mature B lymphoid antigens) but harbored *TCF3-HLF* fusion genes. The discrepant results obtained from different diagnostic tests (eg, morphologic interpretations, immunophenotyping, and karyotype) emphasized the significance of molecular genetic analysis and its crucial role in subgroup classification and prognostication.

*TCF3*, located at 19p, is rearranged with several genes in childhood ALL. A t(1;19) (q23;p13) translocation and *TCF3-PBX1* fusion account for 5% of childhood ALL. Among reported BL-like ALL cases, only t(1;19) is repeatedly found in gene analyses, indicating that it plays a critical role in ALL resembling BL.^3^ The phenotype is also associated with approximately 85% event-free 5-year survival rates.^7,8^ By contrast, childhood ALL harboring t(17;19) (q22;p13) translocation and *TCF3-HLF* fusion is extremely rare (< 0.5% of cases) but very aggressive and associated with a poor outcome.^8-10^

Recent next-generation sequencing studies on the pathogenesis of BL have revealed that mutations in the transcription factor TCF3 or its negative regulator ID3 occurred in approximately 70% of sporadic and immunodeficiency-related BL cases and 40% of endemic BL cases. TCF3 promotes survival and proliferation in lymphoid cells by activating the B-cell receptor/phosphatidylinositol 3-kinase signaling pathways and modulating the expression of cyclin D3, which is also mutated in 30% of BL.^11,12^

The Berlin-Frankfurt-Munich protocol for pediatric Burkitt lymphoma/leukemia is promising. Few patients (7.9%; 157/1,979) demonstrate disease progression or relapse.^13^ Consistent with the World Health Organization consensus, which classified BL as a leukemic variant of Burkitt lymphoma, the TPOG treats patients with BL using the TPOG 10 B-NHL-R4 protocol.^5^ By contrast, patients with other forms of B-cell ALL are treated using the ALL protocol.^14^ However, *TCF3-HLF*-positive ALL is highly resistant to conventional chemotherapy and has a high rate of treatment failure despite treatment intensification and hematopoietic stem cell transplantation.^6^

Molecular tests are becoming increasingly valuable in disease diagnosis, and genetic markers are widely used for subtype classification and prognostication. Detection of *TCF3* rearrangement is crucial in diagnosing ALL, and distinguishing different *TCF3* fusion genes is essential in risk stratification. However, conventional cytogenetic analysis may fail to detect t(8;14) or t(17;19) and underestimate cryptic fusion genes. The MLPA telomere kit circumvents this pitfall because of its higher sensitivity in detecting aneuploidy. Furthermore, it has a shorter turnaround time and higher sensitivity than conventional cytogenetics analysis, irrespective of the mitotic index.^15,16^

This case report highlights the relevance of discrepant diagnostics, the importance of genetic marker identification, and the need for molecular test development and application. MLPA aids in the identification of copy-number variations and aneuploidy, which may be missed in conventional cytogenetic analysis. Thus, it may be a reasonable supplement to routine diagnostics in patients with childhood B-ALL, especially those with unknown subtypes of B-cell ALL and without metaphases or normal karyotype.

## Figures and Tables

**TABLE A1.**
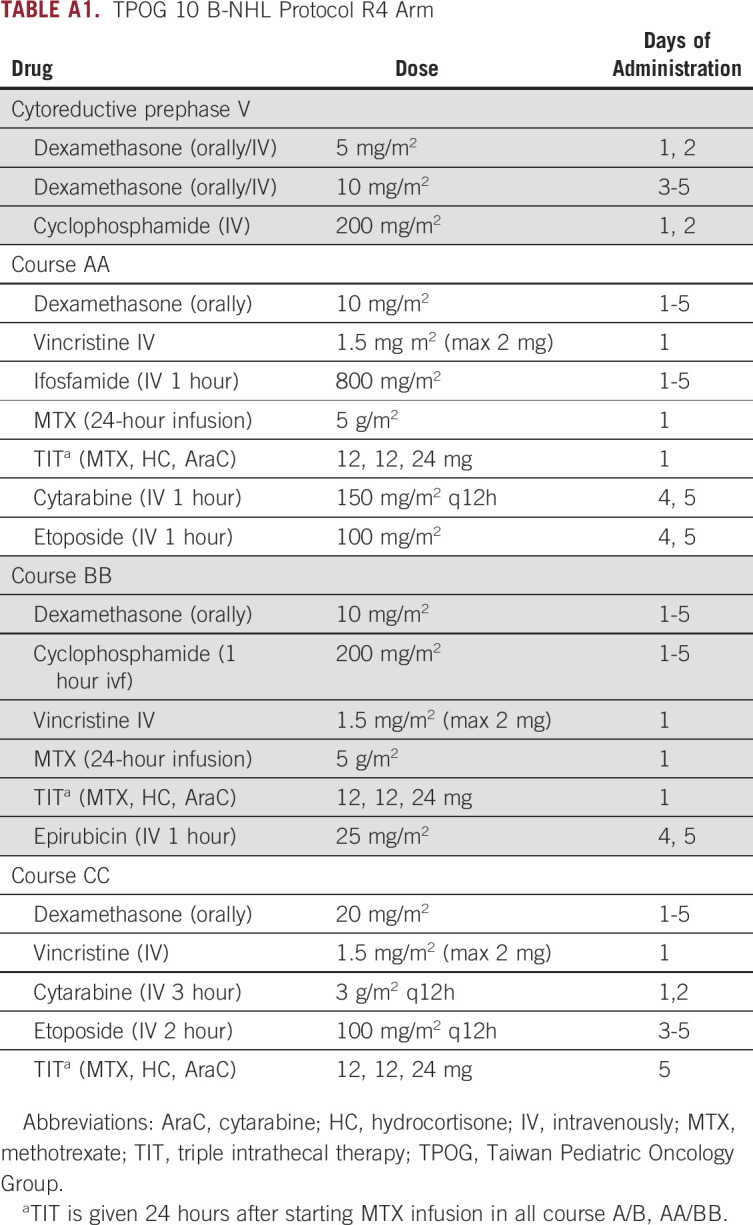
TPOG 10 B-NHL Protocol R4 Arm

## Data Availability

The data from the study will be made available by the authors on request.
